# Inoculating moldavian balm (*Dracocephalum moldavica* L.) with mycorrhizal fungi and bacteria may mitigate the adverse effects of water stress

**DOI:** 10.1038/s41598-023-43539-3

**Published:** 2023-09-27

**Authors:** Rouhollah Amini, Parisa Zafarani-Moattar, Mohammad Reza Shakiba, Alireza Hasanfard

**Affiliations:** 1 Department of Plant Ecophysiology, Faculty of Agriculture, University of Tabriz, Tabriz, Iran; 2 Department of Agrotechnology, Faculty of Agriculture, Ferdowsi University of Mashhad, Mashhad, Iran

**Keywords:** Plant physiology, Plant stress responses

## Abstract

Plant growth-promoting bacteria (PGPBs) play a crucial role in mitigating the oxidative damage caused by water stress in different plant species. The aim of this study was to determine the effects of PGPBs and mycorrhiza-like fungi (*Piriformospora indica*) on improving drought tolerance in moldavian balm (*Dracocephalum moldavica* L.), a medicinal and aromatic plant. For this purpose, a greenhouse study was conducted in a factorial experiment based on a randomized complete design with three replications. Results indicate that water stress reduces the membrane stability index (MSI), total chlorophyll content (Chlt), carotenoids, and maximum photochemical efficiency of photosystem II (Fv′/Fm′) in moldavian balm plants, while increasing superoxide dismutase (SOD) activity, malondialdehyde (MDA) content, and hydrogen peroxide (H_2_O_2_) content compared to the control (no water stress). Inoculation with PGPBs and *Piriformospora indica* helped alleviate the negative effects of water stress. The highest MSI (48%) and Fv′/Fm′ value (0.82) were observed when inoculated with *Enterobacter* and *Piriformospora*, respectively, under non-water-stressed conditions. Inoculation with *Agrobacterium*, *Piriformospora*, and *Enterobacter* improved the Chlt and leaf proline contents, as well as the SOD activity under high water stress, compared to the non-inoculated control values. Furthermore, inoculation with *Pseudomonas* under high water deficit stress levels increased the MDA content (0.51 mmol g^−1^ FW) and H_2_O_2_ levels (0.40 mmol g^−1^ FW). The highest yield of flowering branches (2.414 g pot^−1^) in moldavian balm was obtained with *Enterobacter*. Based on the enhanced physiological and biochemical responses, as well as increased antioxidant enzyme activity that improve water tolerance in this plant, it is recommended to use PGPBs and *Piriformospora indica* fertilization.

## Introduction

Moldavian balm (*Dracocephalum moldavica* L.) is an annual herbaceous plant that belongs to the Lamiaceae family. It is known for its production of essential oils and its medicinal-aromatic properties. The moldavian balm is native to the temperate climate of Asia but has been naturalized in Eastern and Central Europe, North Africa, China, and the northeastern United States. This plant is distributed in the northwest of Iran, including Tabriz, Urmia, Mazandaran, and the Alborz mountain ranges^1^. This annual plant is 25–75 cm tall and has numerous stems and blue flowers arranged in pseudo-whorls, growing in leaf axils. The essential oil of moldavian balm accumulates in the leaves, inflorescence, and stems in oil-containing cells^1^. Moldavian balm's essential oil is used for food, cosmetics, flavorings, and pharmaceutical purposes. The aerial part of moldavian balm is used in Iran for its stomachic, digestive, sedative, diaphoretic, and medicinal properties.

Water deficit stress reduces the availability and uptake of nutrients, which may lead to a decrease in transpiration, stomatal conductance, photosynthetic rate, and growth^2^. Water deficit stress eventually leads to desiccation stress, decreasing leaf water potential and relative water content (RWC). A significant reduction in chlorophyll content can be observed due to water stress^3^. The osmotic adjustment, mainly through proline accumulation, is a typical response of crops to environmental stresses^4^. This decreases the osmotic potential and maintains cell turgor pressure at a high level, enabling plants to sustain their physiological processes. The reactive oxygen species (ROS) level can also increase in plants due to water deficit stress. ROS can disrupt normal cellular activities when it reaches the threshold level, potentially leading to increased membrane lipid peroxidation^5^. Then, the breakdown of fatty acids could result in the formation of small hydrocarbon fragments, including malondialdehyde (MDA)^6^.

Antioxidant enzymes, such as superoxide dismutase (SOD) and peroxidase (POD), help plants protect cellular and sub-cellular systems from the cytotoxic effects of active oxygen radicals^6^. Helal and Samir^7^ found that modulating the activities of these enzymes can play a critical role in imparting plant resistance against water deficit stress. In medicinal plants, water deficit stress can also reduce solute and antioxidant accumulation^8^. Solinas et al.^9^ reported that water deficit stress is a significant factor that affects the synthesis of natural products, thereby altering the secondary metabolites in plants.

Plant physiology is directly affected by certain bacterial strains, which can increase the synthesis of plant hormones. In various crops, plant growth-promoting bacteria (PGPBs) play a crucial role in mitigating oxidative damage caused by abiotic stress by manipulating antioxidant enzymes^10^. Under water deficit stress conditions, inoculation with *Piriformospora indica* enhanced the activity of antioxidant enzymes, increased the levels of osmolytes, and protected the chlorophyll pigments^11^. In cropping systems, inoculation with *Piriformospora indica* (a mycorrhiza-like fungus)^12^ and PGPBs^13^ is a suitable strategy to mitigate the effects of water deficit stress. Inoculation with PGPBs improved root elongation, reduced the effects of water deficit stress under field and greenhouse conditions, enhanced nutrient mobility, and ultimately improved crop growth and production^14^. Inoculation with arbuscular mycorrhizal fungi (*Glomus versiforme*) improved the growth and herbage yield of moldavian balm under water stress^15^. The yield of Moldavian balm herbage decreased by 45% under high water deficit stress compared to the no stress treatment^16^.

Using PGPBs and *Piriformospora indica* can enhance certain physiological parameters, the activity of antioxidant enzymes, and the production of secondary metabolites in moldavian balm^10,11^. In addition, their use leads to a reduction in the use of chemical fertilizers in agroecosystems. Based on the documentation, there is no available information regarding the impact of PGPBs and *Piriformospora indica* on mitigating the adverse effects of water stress in this species. Therefore, this study was conducted for the first time with the aim of evaluating the effect of these compounds on specific biochemical and physiological attributes that contribute to drought tolerance and adaptation in moldavian balm.

## Materials and methods

### Experimental treatments

The study was conducted twice, once in 2018 and again in 2019, in a research greenhouse at the University of Tabriz in Iran. The total duration of the experiment, from sowing to harvesting, was 92 days in both years. The two-factor factorial experiment was arranged as randomized complete block design (RCBD) with three replications. The factors were water deficit stress at four levels based on field capacity (FC) including no water deficit stress (85–100% FC), low water deficit stress (70–85% FC), medium water deficit stress (55–70% FC), high water deficit stress (40–55% FC) and inoculation levels consisted of control (non-inoculated), inoculation with *Agrobacterium* sp., *Pseudomonas fluorescens*, *Piriformospora indica* and *Enterobacter cloacae*.

### Preparing the mycorrhiza-like fungi and bacterial strains

The bacterial strains consisted of *Agrobacterium* sp. 14A-4, *Enterobacter* S16-3 and *Pseudomnas* sp. C16-2O were prepared in the soil biology laboratory, University of Tabriz, Iran. The bacterial strains (an overnight culture in 16 h) prepared in nutrient broth (NB) and after reaching the desired optical density was applied in the study. In the pot experiment the bagasse (sterilized): perlite (50:50) was used as a carrier. The mixture's moisture reached to 20% by adding distilled water, and then bacterial suspension (10 ml) containing 10^8^ Colony-Forming Unit (CFU) ml^−1^ was added to the 100 g bagasse: perlite mixture and applied as inoculum. The NB medium contained beef extract 10 g, NaCl 5 g L^−1^, and peptone 10 g^17^.

The *Piriformospora indica* fungi was provided from the collection of Soil Biology Laboratory, University of Tabriz, Iran. The *Piriformospora indica* was propagated on a plate in Kafer medium for 2 weeks under 24 °C^17^. After propagation, the mycelium of *Piriformospora* was added to the sterilized bagasse:perlite (50:50) carrier and applied as inoculum treatment in the experiment. The prepared inoculum was contained mycelium and of 10^4^ spores g^−1^ of *Piriformospora indica.*

### Moldavian balm pots management

The plastic pots with 25 cm diameter and 30 cm height containing 6 kg soil were used for the experiment. The pot's soil was provided from the University of Tabriz research field, which was sampled at a depth of 0–20 cm. The physiochemical characteristics of the soil are presented in Table 1. For the nutrient requirement of moldavian balm, 0.9 g urea, 0.15 g potassium sulphate and 0.15 g triple superphosphate were used in experimental pots (6 kg of soil). To remove the effect of indigenous microorganisms on inoculation treatments, the soil of pots was sterilized at 120 °C, 1 atm for 45 min before use in the experiment. The prepared inoculums of *Agrobacterium*,* Pseudomonas*,* Piriformospora indica*, and *Enterobacter* were added to the soil of the pots before moldavian balm sowing at rates of 8.0, 8.0, 10.0 and 8.0 g pot^−1^, respectively. After inoculation with *Piriformospora indica* and bacterial strains the soil surface in the pots was fog sprayed. No inoculum was used in control.

**Table 1:** Physico-chemical properties of the soil used in the experiment.

Parameter	Value
Soil texture	Sandy loam
Clay (%)	15
Silt (%)	26
Sand (%)	59
Field capacity (%)	19.0
pH	7.80
OC (%)	0.91
EC (dS m^−1^)	2.10
N (%)	0.30
P (mg kg^−1^)	13.0
K (mg kg^−1^)	326.0

Seeds of moldavian balm were purchased from Pakan-Bazr Isfahan, Iran. In all pots, ten seeds of moldavian balm were sown at the 1-cm soil depth and after seedling emergence and establishment, five plants were kept in each pot and the others were removed. Three pots were considered for all treatments. The moldavian balm pots were placed in a greenhouse with a relative humidity of 60–70% and a temperature of 22 ± 2 °C. The FC of the soil was determined using a pressure plate apparatus. The irrigation of pots was done by the weighing method based on water deficit stress levels to reach the desired FC level as needed. Water deficit stress levels were exerted from the 4–5-leaf stage of moldavian balm seedlings to the maturity of plants.

### Measurements of physiological and biochemical traits

At 50% flowering stage, water stress injury to the cell membrane was measured by electrolyte leakage (EL) using an electrical conductivity meter (Jenway Model 4510, UK). The membrane stability index (MSI) was calculated using Eq. (1) and (2)^18^:

1 $$\mathrm{EL} (\%)=(\mathrm{EC}1/\mathrm{EC}2)\times 100$$

2 $$\mathrm{MSI}=1-\mathrm{EL\%}$$

where EC1 is the initial conductivity, EC2 is the conductivity of the killed samples (using autoclave 110℃ at a pressure of 1.2 atm for 30 min).

RWC according to Teulat et al.^19^, Chlt contents and carotenoid contents based on Arnon^20^, Maximum photochemical efficiency of photosystem II (Fv′/Fm′) according to the Maxwell and Johnson^21^, water soluble carbohydrates (WSCs) using the method of Kochert^22^, POD as proposed by Ghanati et al.^23^, SOD as reported by Zhang et al.^24^, leaf proline (LP) content according to the Bates et al.^25^, MDA content as mentioned by Li^26^ and H_2_O_2_ was measured according to Alexiva et al.^27^. Leaf soluble protein (SP) content was also evaluated by the method of Bradford^28^. A sample of the plants (five) in each pot was taken to measure the mentioned attributes. To measure flowering branches’ yield, 25 cm above the branches were cut and their weight was recorded after they dried.

### Statistical analysis

The analysis of variance (ANOVA) was done as a two-factor factorial experiment (4 × 5) based on RCBD with three replications by SAS software ver. 9.4 (SAS Institute, Inc.; Cary, NC, USA). The experiment was carried out twice and the data of both experiments were pooled for analysis because the time × treatment interaction was not significant. The data of moldavian balm physiological and biochemical traits were used in ANOVA passed the tests of normality and homogeneity of variance. The Duncan multiple range test was used for mean comparison at P ≤ 0.05.

### Ethical approval

The authors confirm that the use of plants in the present study complies with international, national, and institutional guidelines.

## Results

### Membrane stability index (MSI)

**Figure 1:**
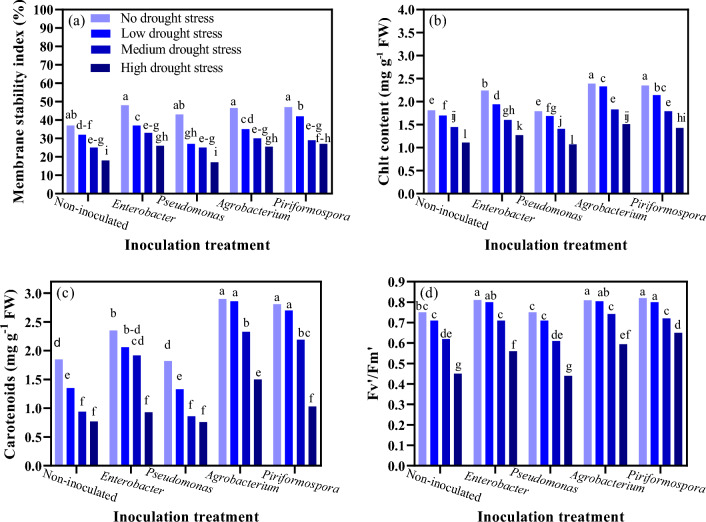
The effect of inoculation treatment and water deficit stress on membrane stability index (**a**), total chlorophyll (Chlt) (**b**), carotenoids (**c**), maximum photochemical efficiency of photosystem II (Fv′/Fm′) (**d**) of moldavian balm. The means with a similar letter are not significantly different at the 1% probability level. No water deficit stress: 85–100% FC; low water deficit stress: 70–85% FC; medium water deficit stress: 55–70% FC; high water deficit stress: 40–55% FC.

Results of statistical analysis showed that MSI was significantly affected by the interaction of water deficit stress and inoculation (P ≤ 0.01). The highest MSI (48%) was observed in the inoculation with *Enterobacter* in control conditions (85–100% FC) (Fig. 1a). This treatment was not significantly different from other inoculation treatments at this stress level. The lowest MSI (14%) was observed in the inoculation with *Pseudomonas* under high water deficit stress (40–55% FC) (Fig. 1a).

### Total chlorophyll (Chlt) content

Based on ANOVA results, the interaction of water deficit stress and inoculation was significant on Chlt content (P ≤ 0.01). The mean comparison (Fig. 1b) showed that increasing the water deficit stress to a high level significantly decreased the Chlt content of moldavian balm in all inoculation treatments. The reduction in Chlt content of moldavian balm under high water deficit stress compared to the no water deficit stress level were 39.43%, 40.22%, 36.82%, 43.30%, and 38.67% for inoculation with *Agrobacterium*, *Pseudomonas*, *Piriformospora*, *Enterobacter* and control, respectively. Inoculation with *Agrobacterium, Piriformospora* and *Enterobacter* increased the moldavian balm Chlt content under all water deficit stress levels. *Pseudomonas* inoculation had no significant effect on Chlt content of moldavian balm. Under no water deficit stress level, inoculation with *Agrobacterium*, *Piriformospora*, and *Enterobacter* enhanced the moldavian balm Chlt content by 24.26%, 22.97%, and 19.19%, respectively, compared with control. Also under high water deficit stress level, inoculation with *Agrobacterium*, *Piriformospora*, and *Enterobacter* enhanced the moldavian balm Chlt content by 26.49%, 22.37%, and 12.59%, respectively, in comparison with control (Fig. 1b). The highest Chlt content of moldavian balm (2.39 mg g^−1^ FW) was obtained under no water deficit stress level and inoculation with *Piriformospora* and *Agrobacterium* (2.35 mg g^−1^ FW). Generally, inoculation with *Agrobacterium* had the highest Chlt content under all water deficit stress levels compared with those in other inoculation levels. There was no significant difference among the inoculation with *Piriformospora* and *Agrobacterium* under all water deficit stress levels.

### Carotenoid content

According to the ANOVA, water deficit stress and inoculation significantly influenced carotenoid content (P ≤ 0.01). As shown in Fig. 1c, the highest carotenoid content (2.9 mg g^−1^ FW) was obtained in the inoculation with *Agrobacterium* in control conditions (85–100% FC). At this stress level, no significant difference was observed between inoculation with *Agrobacterium* and *Piriformospora*. The lowest carotenoid content (0.76 mg g^−1^ FW) was observed in the inoculation with *Pseudomonas* under high water deficit stress (40–55% FC). However, no significant difference was observed between inoculation with *Pseudomonas*, *Piriformospora*, *Enterobacter*, and non-inoculated control at this stress level (Fig. 1c).

### Fv′/Fm′ value

The interaction of water deficit stress with inoculation significantly affected Fv′/Fm′ value in moldavian balm (P ≤ 0.05). Data presented in Fig. 1d showed that the highest Fv′/Fm′ value (0.82) was obtained in inoculation with *Piriformospora* in conditions without water stress. However, there was no significant difference between inoculation with this treatment and other treatments at this stress level. The lowest Fv′/Fm′ value (0.44) was observed in the inoculation with *Pseudomonas* under high water deficit stress (40–55% FC). Furthermore, no significant difference was observed between inoculation with *Pseudomonas* and non-inoculated control in this stress level.

### Water soluble carbohydrates (WSCs)

**Figure 2:**
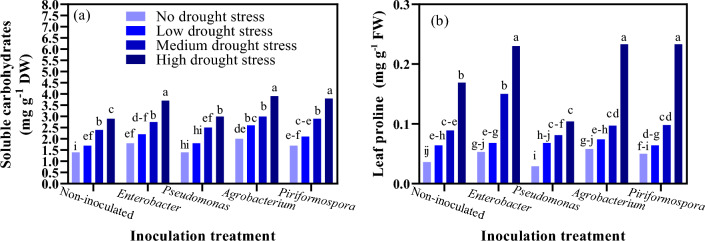
The effect of inoculation treatment and water deficit stress on soluble carbohydrates (**a**) and leaf proline (**b**) of moldavian balm. The means with a similar letter are not significantly different at the 1% probability level. No water deficit stress: 85–100% FC; low water deficit stress: 70–85% FC; medium water deficit stress: 55–70% FC; high water deficit stress: 40–55% FC.

Interaction of water deficit stress with inoculation had a significant effect on changes in WSCs in moldavian balm (P ≤ 0.01). The highest WSCs (3.9 mg g^−1^ DW) in inoculated with *Piriformospora* under high water stress (40–55% FC) and its lowest (1.42 mg g^−1^ DW) in the non-inoculated control and the condition without stress (85–100% FC) were observed (Fig. 2a).

### Leaf proline (LP) content

The mean comparison of interaction indicated that in control, increasing the water deficit stress level from no to high water deficit stress level increased the LP content in moldavian balm significantly (Fig. 2b). In *Enterobacter*, *Piriformospora* and *Agrobacterium* inoculation treatments, the LP contents under high water deficit stress level significantly increased (36.09%, 37.86%, and 37.45%, respectively) in comparison with that in control (Fig. 2b). The greatest LP content (0.23 mg g^−1^ FW) was observed in inoculation with *Piriformospora*, *Agrobacterium*, and *Enterobacter* under high water deficit stress levels. However, moldavian balm LP content was not affected by inoculation with *Pseudomonas*.

### Relative water content (RWC)

Water deficit stress and inoculation treatment significantly affected RWC in moldavian balm (P ≤ 0.01). However, the interaction of water deficit stress with inoculation did not affect RWC. The mean comparison results indicated that the no water deficit stress had the highest RWC (71.56%) and the lowest one was observed under a high water deficit stress level (46.7%). Inoculation with *Piriformospora* and *Pseudomonas* had the most significant and lowest RWC (64.78% and 48.84%), respectively (Table 2). The RWC in *Pseudomonas* inoculation was not significantly different from that in control.

**Table 2:** The effect of water deficit stress and inoculation treatments on moldavian balm relative water content (RWC), peroxidase (POD) activity, and leaf soluble protein (SP) content.

Treatment	RWC (%)	POD activity (μg^−1^ FW)	Leaf SP content (mg g^−1^ DW)
Water deficit			
No water deficit stress	71.56^a^	0.3159^b^	0.7126^c^
Low water deficit stress	61.73^b^	0.3475^b^	0.7767^c^
Medium water deficit stress	57.61^b^	0.3983^a^	0.8890^b^
High water deficit stress	46.69^c^	0.4308^a^	1.1930^a^
Inoculation			
Non-inoculated control	55.75^b^	0.2945^b^	0.7476^b^
*Enterobacter*	64.54^a^	0.4825^a^	1.007^a^
*Pseudomonas*	48.84^b^	0.2146^b^	0.7346^b^
*Agrobacterium*	63.08^a^	0.4028^a^	1.016^a^
*Piriformospora indica*	64.78^a^	0.4512^a^	0.9596^a^

Means within each column with similar letter are not significantly different at the 1% probability level. No water deficit stress: 85–100% FC; low water deficit stress: 70–85% FC; medium water deficit stress: 55–70% FC; high water deficit stress: 40–55% FC.

### Peroxidase (POD) activity

The mean comparison of water deficit stress levels showed that the high water deficit stress level had the greatest POD activity (0.4308 μg^−1^ FW), and the lowest one (0.3159 μg^−1^ FW) was observed under no water deficit stress level (Table 2). Among the inoculation treatments, the highest POD activity (0.4825 μg^−1^ FW) was observed in *Enterobacter* treatment that was not significantly different with those in inoculation with *Agrobacterium* and *Piriformospora*. The lowest POD activity (0.2146 μg^−1^ FW) was observed in inoculation with *Pseudomonas* which had no significant difference with that in control (Table 2).

### Superoxide dismutase (SOD) activity

**Figure 3:**
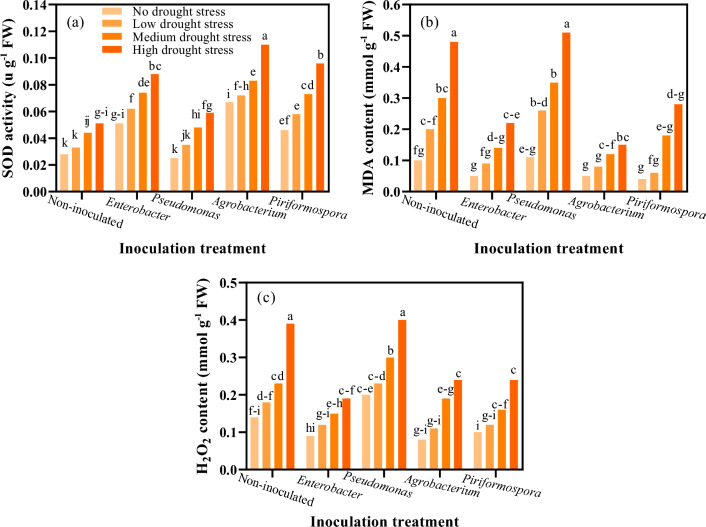
The effect of inoculation treatment and water deficit stress on superoxide dismutase (SOD) activity (**a**), malondialdehyde (MDA) content (**b**), and hydrogen peroxide (H_2_O_2_) content (**c**) of moldavian balm. The means with a similar letter are not significantly different at the 1% probability level. No water deficit stress: 85–100% FC; low water deficit stress: 70–85% FC; medium water deficit stress: 55–70% FC; high water deficit stress: 40–55% FC.

Interaction of water deficit stress with inoculation significantly affected SOD activity in moldavian balm (P ≤ 0.01). Results indicate that in all inoculation treatments, the activity of SOD increased as the level of water deficit stress increased (Fig. 3a). The increase in SOD activity under high water deficit stress (40–55% FC) compared with no water deficit stress level (85–100% FC) was 39.09%, 57.62%, 52.08%, 42.04% and 45.09% for inoculation with *Agrobacterium*, *Pseudomonas, Piriformospora*, *Enterobacter*, and control (non-inoculated), respectively. The SOD activity was not affected by inoculation with *Pseudomonas,* while under all water deficit stress levels, inoculation of *Agrobacterium*, *Piriformospora* and *Enterobacter* increased the SOD activity. Inoculation with *Agrobacterium*, *Piriformospora* and *Enterobacter* increased the SOD activity by 53.63%, 46.0%, and 42.04%, compared with that in control, under high water deficit stress (Fig. 3a). The highest SOD activity (0.11 μg^−1^ FW) was observed in inoculation with *Agrobacterium* under high water deficit stress. The lowest SOD activities were observed in inoculation with *Pseudomonas* which had no significant difference from those in control.

### Malondialdehyde (MDA) content

The results of mean comparison for interaction showed that in all inoculation treatments the moldavian balm MDA content increased with increasing the water deficit stress level (Fig. 3b). The highest MDA content (0.51 mmol g^−1^ FW) was observed in inoculation with *Pseudomonas* under high water deficit stress level that was not significantly different with that in control under high water deficit stress level. Inoculation with *Pseudomonas* under high water deficit stress resulted in an 80% increase in MDA levels compared to non-inoculated plants without water stress.

### Hydrogene peroxide (H_2_O_2_) content

The results of the mean comparison for H_2_O_2_ content (Fig. 3c) showed that in all inoculation treatments, by increasing the water deficit stress level, the H_2_O_2_ content increased significantly. Among the inoculation treatments, the highest H_2_O_2_ contents were observed in non-inoculated and *Pseudomonas* treatments. The highest content of H_2_O_2_ (0.4 mmol g^−1^ FW) was observed in inoculation with *Pseudomonas* under high water deficit stress level which was not significantly different from control (non-inoculated) under high water deficit stress level (Fig. 3c). Generally inoculation with *Agrobacterium*, *Piriformospora* and *Enterobacter* significantly decreased the H_2_O_2_ content under all water deficit stress levels, in comparison from control. Under low and high water deficit stress levels, inoculation with *Agrobacterium* significantly decreased the H_2_O_2_ content by 38.88% and 38.46% respectively, in comparison with that in control.

### Leaf soluble protein (SP) content

Water deficit stress and inoculation treatment significantly affected SP content in moldavian balm (P ≤ 0.01). However, the interaction of water deficit stress with inoculation did not affect SP content. The mean comparison results indicated that the high water deficit stress had the highest SP content (1.19 mg g^−1^ DW), and the lowest was obtained under no water deficit stress (0.71 mg g^−1^ DW). Inoculation with *Agrobacterium*, *Enterobacter*, and *Piriformospora* had the highest SP content (1.01, 1.00, and 0.96 mg g^−1^ DW, respectively) (Table 2).

### Flowering branches yield (FBY)

**Figure 4:**
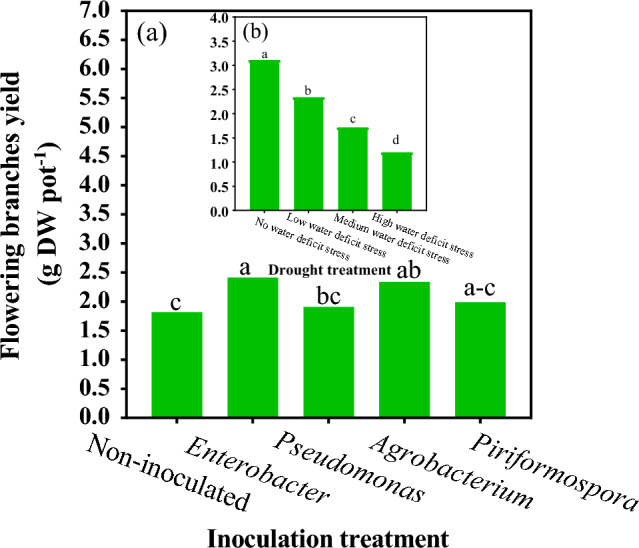
The effect of water deficit stress and inoculation treatments on flowering branches yield of moldavian balm. The means with a similar letter are not significantly different at the 1% probability level. No water deficit stress: 85–100% FC; low water deficit stress: 70–85% FC; medium water deficit stress: 55–70% FC; high water deficit stress: 40–55% FC.

Water deficit stress (P ≤ 0.01) and inoculation treatment (P ≤ 0.05) significantly affected flowering branches yield. However, the interaction of water deficit stress with inoculation did not affect flowering branches yield. Among the inoculation treatments, the highest FBY (2.414 g pot^−1^) was obtained in *Enterobacter*, which was not significantly different from those in inoculation with *Agrobacterium* and *Piriformospora* (Fig. 4a). No water deficit stress had the highest FBY (3.111 g pot^−1^), and the lowest one (1.201 g.pot^−1^) was observed under high water deficit stress (Fig. 4b).

## Discussion

For the first time, this study investigated the effects of PGPBs and *Piriformospora indica* on mitigating water stress in moldavian balm. For this purpose, the physiological and biochemical responses of the plant to these compounds and water stress levels are discussed.

MSI is considered one of the important selection criteria for assessing plant drought tolerance. Membrane lipids underwent significant changes in moldavian balm when it experienced water stress (Fig. 1a). Subsequently, the increase in free radicals and, as a result, cell destruction has caused oxidative stress. PGPBs and *Piriformospora indica* likely enhance cell membrane integrity by activating genes associated with water stress tolerance and altering proteins involved in energy metabolism. The levels of Chlt and carotenoid content in moldavian balm were reduced by increasing water deficit stress levels under all inoculation treatments. However, inoculation with *Piriformospora*, *Agrobacterium*, and *Enterobacter* increased the content under all water deficit stress levels compared to *Pseudomonas* inoculation (Fig. 1b,c). In this study, inoculation with these compounds likely increased the chlorophyll content of moldavian balm under water stress by providing more absorption sites for nutrients and water. Water stress often limits the availability of nutrients in the soil, leading to nutrient deficiencies in plants. In this study, it is likely that beneficial bacteria enhance the solubilization and absorption of nutrients by colonizing the plant's root system, thereby forming a symbiotic relationship. This process of colonization ensures a sufficient supply of essential nutrients for the synthesis of chlorophyll. Additionally, the application of bacteria can induce systemic resistance in plants, activating their defense mechanisms against water stress. This can result in improved water-use efficiency and reduced oxidative damage, which can have a positive impact on chlorophyll content. Das et al.^29^ reported that inoculation with *Piriformospora indica* in coleus (*Plectranthus barbatus*) increased the total chlorophyll content of the leaves by 30% compared to the non-inoculated treatment. In another experiment, it has been reported that inoculating *Solanum melongena* L. cultivars with *Piriformospora indica* reduced the adverse effects of drought stress on total chlorophyll levels^30^.

The Fv′/Fm′ parameter indicates the maximum quantum yield of photosystem II and is used for early water stress detection in plants. Moldavian balm inoculated with *Piriformospora* had a higher Fv′/Fm′ value than the other treatments (Fig. 1d). Therefore, applying this treatment has helped maintain the chlorophyll content and electron transfer cycle during periods of water stress. In other words, the application of *Piriformospora* has increased the efficiency of converting light energy and the potential activity of photosynthetic reaction centers by increasing Fv′/Fm′.

Improved WSCs in plants treated with PGPBs and *Piriformospora indica* may be attributed to increased α-amylase activity, resulting in starch breakdown (Fig. 2a). Probably, these compounds play a role as osmotic regulators in the treated plants. Among the inoculation treatments, the lowest LP contents were observed in the treatment with *Pseudomonas* (Fig. 2b). Under high water deficit stress, inoculation with *Enterobacter*, *Agrobacterium*, and *Piriformospora* significantly increased the LP contents compared to inoculation with *Pseudomonas* and the control. Inoculation with PGPBs and *Piriformospora indica* increased the content of LP by making mineral elements available for plant uptake. Zarea et al.^31^ reported that under salinity stress, inoculating wheat (*Triticum aestivum* L.) with *Piriformospora indica* and *Azospirillum* improved the proline content and plant growth.

The RWC of moldavian balm was significantly reduced by increasing the level of water deficit stress. Similar results have also been reported by Alaei et al.^32^ in moldavian balm and Kazeminasab et al.^33^ in lemon balm (*Melissa officinalis* L.). Inoculation with *Piriformospora*, *Agrobacterium*, and *Enterobacter* increased the RWC of moldavian balm. Hosseini et al.^34^ also observed that wheat plants colonized with *Piriformospora indica* had higher RWC under stress conditions. Similarly, Ahmad et al.^35^ reported that inoculation with *Enterobacter* did not affect the RWC of maize (*Zea mays* L.).

The POD activity was significantly enhanced by increasing the level of water deficit stress. Gusain et al.^36^ also reported that in rice (*Oryza sativa* L.), the activity of POD increased under water stress conditions. Increasing the activity of POD can be attributed to the excessive accumulation of ROS in plant cells under conditions of water deficit stress. Inoculation with *Agrobacterium*, *Piriformospora*, and *Enterobacter* increased the POD activity of moldavian balm compared to that of the control. Saravanakumar et al.^37^ also reported that in green gram (*Vigna radiata* L.), the activities of POD in plants inoculated with *Bacillus subtilis* and *Pseudomonas fluorescens* increased significantly compared to non-inoculated plants. Furthermore, inoculation with *Dracocephalum moldavica* with *Micrococcus yunnanensis* (PGPB) and *Claroideoglomus etunicatum* (an arbuscular mycorrhizal fungus) improved the antioxidant activity, including POD enzyme, which can mitigate the detrimental effects of water deficit stress^38^.

The SOD activity of moldavian balm was enhanced by increasing the level of water deficit stress in all inoculation treatments. Inoculation with *Agrobacterium* resulted in the highest SOD activity among all the inoculation treatments under different water deficit stress levels (Fig. 3a). SOD, an antioxidant enzyme in chloroplasts, increases protection against oxidative stress^6^. Using the PGPBs can prevent oxidative damage by increasing SOD activity. These results are consistent with the findings of Galicia-Campos et al.^39^ regarding the beneficial effects of PGPBs.

The MDA content increased in all inoculation treatments, except for the *Agrobacterium* treatment, as the level of water deficit stress increased. Inoculation with *Pseudomonas* resulted in the highest MDA content under conditions of high water deficit stress. This result could be attributed to the high accumulation of H_2_O_2_ in the cells during the treatment. One of the consequences of H_2_O_2_ accumulation is the production of MDA and the peroxidation of membrane lipids. The lowest levels of MDA were observed in the inoculation treatments with *Enterobacter* and *Agrobacterium*, compared to the other treatments. Qiusheng et al.^40^ also reported similar results by inoculating peanuts (*Arachis hypogaea*) with *Agrobacterium tumefaciens*. El-Tayeb^41^ observed that the *Phaseolus acutifolius* plants that were inoculated showed a decrease in MDA content compared to the control plants (*Vicia faba* L.). This decrease in MDA content led to an increase in resistance to prolonged water deficit stress.

The H_2_O_2_ content increased significantly in all inoculation treatments as the level of water deficit stress increased. Inoculation with *Pseudomonas* resulted in higher H_2_O_2_ content under all levels of water deficit stress compared to the other inoculation treatments. Generally, inoculation with *Pseudomonas* had no significant effect on the reduction of H_2_O_2_ accumulation under water deficit stress conditions. Inoculation treatments, such as *Agrobacterium*, *Piriformospora*, and *Enterobacter*, significantly reduced the H_2_O_2_ contents under all levels of water deficit stress. Gusain et al.^36^ also reported that the H_2_O_2_ content increased under high water deficit stress levels, and the inoculation of rice with PGPBs reduced the accumulation of H_2_O_2_. Inoculation with PGPBs led to a reduction in ROS levels under water deficit stress conditions. Therefore, these microorganisms prevent the harmful effects of H_2_O_2_ on plant growth and development under conditions of water deficit stress.

One of the defense mechanisms during osmotic stress is the synthesis of proteins. Accumulation of ROS in the cell leads to the activation of specific signaling pathways that regulate gene expression, modifications in protein synthesis and activity, and ultimately prepares the cell to adapt to new conditions^42^. In this way, the increase in protein content resulting from stress and inoculation with these compounds has led to the regulation of plant osmosis. Nadeem et al.^43^ reported that inoculation with *Agrobacterium* under stress conditions helps to preserve soluble leaf proteins in crop plants. The use of *Enterobacter* has likely resulted in an increase in the yield of flowering branches by enhancing plant tolerance mechanisms to drought, compared to other treatments. Therefore, it is recommended to use Enterobacter to alleviate the detrimental effects of water stress in moldavian balm. Similarly, a study on Calendula (*Calendula officinalis* L.) showed that the application of *Enterobacter* increased the number of flowering branches by providing nitrogen and phosphorus^44^. In general, although the physiological and biochemical response of moldavian balm to the application of the mentioned compounds varied, the positive impact of these compounds on the plant's ability to tolerate water stress was evident. Therefore, if other positive aspects of this species yield favorable results, it is recommended to promote its cultivation in arid and semi-arid regions worldwide, using similar techniques.

## Conclusion

Considering the global challenge of drought, the utilization of compounds that enhance plants' ability to tolerate water stress will play a crucial role in water management. For the first time, this study revealed the tolerance mechanisms of moldavian balm induced by PGPBs and *Piriformospora indica* under water stress. PGPBs, particularly *Enterobacter*, and *Piriformospora indica*, improved drought tolerance in moldavian balm. This was demonstrated by significant increases in physiological responses and the yield of flowering branches under water stress. The application of these compounds improves the activity and levels of enzymatic and non-enzymatic antioxidants, preserves the integrity of the cell membrane, and enhances the efficiency of photosystem II during water stress. This leads to a reduction in the adverse effects of osmotic stress in moldavian balm. Considering the environmental advantages of the mentioned compounds, such as their non-toxicity, natural origin, and biodegradability, they can be considered as suitable alternatives to chemical fertilizers in sustainable agroecosystems. Further research and experimentation are needed to fully explore the potential benefits and mechanisms of inoculating moldavian balm with mycorrhizal fungi and bacteria under water stress conditions. However, these approaches show promise in mitigating the adverse effects of water stress and improving the overall health and productivity of plants.

## Data Availability

The necessary information is available from the corresponding author on reasonable request. The experiments conducted on the studied plant were in compliance with all relevant institutional, national, and international guidelines and legislation.
